# A Cross‐Sectional Study of Platelet‐to‐Lymphocyte Ratio in Relation to Pigment Cell Nevi and Atypical Mole Syndrome

**DOI:** 10.1002/hsr2.70888

**Published:** 2025-06-11

**Authors:** Reetta Nevakivi, Hanna Siiskonen, Salla Haimakainen, Ilkka T. Harvima

**Affiliations:** ^1^ Department of Dermatology University of Eastern Finland and Kuopio University Hospital Kuopio Finland; ^2^ Department of Pathology Kuopio University Hospital Kuopio Finland

**Keywords:** atypical mole syndrome, melanoma, nevus, pigment cell, platelet‐to‐lymphocyte ratio

## Abstract

**Background and Aims:**

Melanocytic nevi (MN) and atypical melanocytic nevi (AMN) are established risk factors for cutaneous malignant melanoma (CMM), with patients exhibiting atypical mole syndrome (AMS) facing an even greater risk. Peripheral blood biomarkers—including neutrophil‐to‐lymphocyte ratio (NLR), eosinophil‐to‐lymphocyte ratio (ELR), platelet‐to‐lymphocyte ratio (PLR), and systemic immune‐inflammation index (SII)—have been linked to various cancers, including CMM. This cross‐sectional study aimed to investigate the relationship between these biomarkers and MN and AMS, which are common phenotypes among melanoma patients.

**Methods:**

A total of 505 subjects (aged 21–79, 254 males and 249 females, 96 with immunosuppression) at elevated risk of any type of skin cancer were examined for MN, AMN, level of cutaneous photodamage, and other potential confounding factors. A peripheral blood sample was analyzed for blood cells.

**Results:**

PLR levels were significantly higher in subjects with more than 50 nevi or AMS. In multivariable logistic regression models, high PLR was significantly associated with having more than 50 melanocytic nevi (OR: 2.015, 95% CI: 1.159–3.501, *p* = 0.01) and with atypical mole syndrome (OR: 4.092, 95% CI: 2.012–8.323, *p* < 0.001). Other significant factors associated with high MN density and AMS were lower age, male sex, higher BMI, no immunosuppression, and fewer actinic keratoses. However, NLR, ELR, and SII showed no relation to MN or AMS.

**Conclusion:**

This study demonstrates a statistically significant association between elevated PLR and the presence of more than 50 MN or AMS, whereas no associations were observed with NLR, ELR, or SII. This specific relationship suggests that PLR may have particular relevance in the context of multiple MN and AMS, potentially influencing its interpretation in melanoma prognosis. However, further studies are needed to validate this finding, explore underlying biological mechanisms, and assess causality.

**Abbreviations**
AK: actinic keratosis
AMN: atypical melanocytic nevus/nevi
AMS: atypical mole syndrome
CBC: complete blood count
CMM: cutaneous malignant melanoma
ELR: eosinophil‐to‐lymphocyte ratio
MN: melanocytic nevus/nevi
NLR: neutrophil‐to‐lymphocyte ratio
PAASI: photoaging area and severity index
PLR: platelet‐to‐lymphocyte ratio
SII: systemic immune‐inflammation index
UVR: ultraviolet radiation

## 1 Introduction

The incidence of cutaneous malignant melanoma (CMM) and other skin cancers has increased in recent decades. Both melanocytic nevi (MN) and atypical melanocytic nevi (AMN) are well‐established risk factors for CMM [1, 2]. While the term AMN is often used interchangeably with histologically dysplastic MN, the correlation between clinical appearance and histologic dysplasia is inconsistent [3, 4, 5, 6, 7]. Therefore, in this context, AMN refers to MN with a clinically atypical appearance and a relatively large size (typically > 5 mm).

The risk of CMM increases with the number of MN and AMN, and is even higher in individuals with atypical mole syndrome (AMS), which can be sporadic or hereditary [8, 9, 10, 11]. The risk is particularly elevated in those with a personal history of CMM and a family history involving two or more relatives, with reported risks up to 1200‐fold [1, 2, 8, 9, 10, 12, 13, 14]. Although definitions of this condition vary, AMS can be characterized by an abundance of MN (over 50), their heterogeneous appearance and distribution across both common and uncommon body sites, along with the presence of clinically atypical nevi [8].

Risk factors for increased MN counts in children largely overlap with those for CMM, including fair skin type, propensity to sunburn, freckling, exposure to ultraviolet radiation (UVR), and a family history of high nevus counts [15, 16, 17]. While the association between immunosuppression and the increased risk for nonmelanoma skin cancer has been widely recognized, especially in organ transplant recipients, the relationship between immunosuppression and the risk of CMM is not as firmly established [18, 19]. Immunosuppressive medications and conditions can increase MN count and may induce dermatoscopic changes [20, 21, 22, 23]. Conversely, a lower number of MN has been detected among patients with atopic dermatitis, possibly due to cutaneous inflammation [24, 25, 26]. These observations suggest that the immune system plays a key role in regulating melanocyte proliferation.

The connection between immunity, inflammation, and carcinogenesis is well‐established [27]. Recently, there has been increasing interest in peripheral blood immunomarkers and their role in cancer prognosis. Particularly, the ratios derived from complete blood count (CBC) components—such as neutrophil‐to‐lymphocyte ratio (NLR), eosinophil‐to‐lymphocyte ratio (ELR), platelet‐to‐lymphocyte ratio (PLR), and systemic immune‐inflammation index (SII)—have shown promising prognostic value and potential to predict treatment efficacy across various cancers, including CMM [28, 29]. Elevated PLR and NLR have been associated with shorter melanoma‐specific survival and higher CMM stages [30]. Additionally, elevated PLR, along with other CBC ratios, has been associated with poorer overall survival and treatment outcomes in CMM patients [31, 32, 33, 34, 35, 36]. These ratios have also been investigated in various dermatological conditions, including psoriasis, atopic dermatitis, vitiligo, and alopecia areata [37, 38, 39, 40].

Given the known CMM risk associated with MN and AMS, along with the role of CBC‐derived biomarkers in both dermatological conditions and CMM prognosis, it is important to examine whether a high number of MN or AMS influences these biomarkers. This study aims to investigate whether PLR, NLR, ELR, and SII differ in patients with numerous nevi or AMS. Understanding baseline alterations in these biomarkers could enhance their prognostic value in melanoma, particularly among patients with multiple MN or AMS—both of which are commonly observed in melanoma patients.

## 2 Methods

### 2.1 Study Subjects

For this study, 505 adult subjects (aged 21–79, males 254, females 249) were recruited at the outpatient clinic of Kuopio University Hospital, Kuopio, Finland. Experienced dermatologists, not involved in the study, reviewed all referrals to the dermatology clinic. They invited patients aged 18 to 80 to participate based on a preliminary risk assessment for skin cancer, which included evaluating the personal history of skin cancers or premalignant lesions, photodamage, number of common and atypical MN, immunosuppression, skin phototype, and family history of melanoma [41]. The study's exclusion criteria included severe psychiatric or neurologic conditions that significantly impaired memory or decision‐making abilities, as well as pregnancy and incarceration. All participants signed informed consent forms and completed a questionnaire on demographic details, personal and family history of skin cancer, other illnesses, medications, UVR exposure, and lifestyle factors, including alcohol use and smoking. Immunosuppressive status was defined by recent or ongoing use of systemic immunosuppressive medications [42]. A total of 96 of the subjects were immunosuppressed, 39 of whom were organ transplant recipients.

Total dermatologic examination of the skin was performed by experienced dermatologists, who counted the number of actinic keratoses (AK) and MN, and assessed photodamage using the PhotoAging Area and Severity Index (PAASI) scoring (range 0–400), as previously described [42]. The subjects were grouped into four categories based on MN count: (1) 0–20; (2) 21–50; (3) 51–100, and (4) more than 100 nevi. AMS was defined by the presence of more than 50 MN and multiple clinically atypical MN. Any past or present skin malignancies were recorded and biopsied if necessary.

A total of 405 subjects had a history of premalignant or malignant skin lesions, either previously diagnosed or detected at their first visit. This included 102 with CMM (16 of whom had melanoma in situ or lentigo maligna and were included in the CMM group), 213 with basal cell carcinoma, and 37 with squamous cell carcinoma. Additionally, 282 subjects had a history of premalignant lesions, including nine cases of Bowen's disease (squamous cell carcinoma in situ) alongside AKs, while the remaining subjects presented with AKs only. Many subjects had multiple types of skin lesions, which have been described in more detail in a recent report [43].

The study was granted approval by the Ethics Committee of Kuopio University Hospital (71/2017), and it adhered to the principles outlined in the Declaration of Helsinki. All data obtained from the study participants were handled in compliance with the General Data Protection Regulation of the European Union.

### 2.2 Blood Tests

A blood sample was collected and analyzed at the Kuopio University Hospital's laboratory (ISLAB) using the Advia 2120i Hematology System (Siemens Healthcare Diagnostics, Eschborn, Germany). Hemoglobin levels were measured photometrically, platelet counts using the light scatter method, and white blood cell and differential counts using the light scatter/peroxidase method. The blood biomarker ratios were calculated as follows: NLR, neutrophil count/lymphocyte count; PLR, platelet count/lymphocyte count; ELR, eosinophil count/lymphocyte count; and SII, (platelet count × neutrophil count)/lymphocyte count [44].

### 2.3 Statistics

Statistical analyses were conducted using IBM SPSS Statistics for Windows, Version 27.0. (Armonk, NY: IBM Corp). Differences between continuous variables were tested using the Mann‐Whitney test for pairwise comparisons and the Kruskal‐Wallis test for multiple group comparisons. Differences between categorical variables were analyzed using the *χ* ^2^ test or Fisher's exact test when the group size was fewer than five members. Correlations between variables were analyzed using Spearman's rank correlation test. The optimal cutoff values for the PLR were calculated using the highest point of Youden's index derived from the receiver operating characteristic (ROC) curves.

Binomial logistic regression analysis was performed to assess the association of various risk factors with nevus count or AMS. Simple odds ratios (ORs) were calculated for each factor. Multivariable models were then formed using variables that produced statistically significant univariate ORs, as well as clinically relevant variables. ROC‐curves and their corresponding area under the curve (AUC) values were used to assess the fit of the models. A *p* value less than 0.05 was considered to be statistically significant. All the tests used were two‐tailed.

## 3 Results

### 3.1 Comparison of Subjects With Different Numbers of Melanocytic Nevi

In a total of 503 subjects with reported nevus counts (Table 1), mean age, PAASI and number of AKs significantly decreased (*p* < 0.001) with increasing number of nevi, when comparing groups with 0–20, 21–50, 51–100, and more than 100 MN. Unexpectedly, the proportion of immunosuppressed subjects decreased as the number of MN increased (23.8%, 18.6%, 11.3%, and 9.8%, respectively, *p* = 0.02). The proportion of subjects with a history of indoor occupation and a family history of CMM increased with more nevi.

**Table 1:** Comparison of all subjects (*n* = 503) by nevus count categories.

	Observed nevus count				*p* value
	0–20 nevi	21–50 nevi	51–100 nevi	Over 100 nevi	
	*n* = 244	*n* = 118	*n* = 80	*n* = 61	
Age					**< 0.001**
Range	29–79	24–79	24–79	21–77	
Mean ± SD	68.09 ± 9.44	60.62 ± 12.65	55.32 ± 16.08	53.28 ± 14.12	
Sex					0.700
Male	117 (48.0)	64 (54.2)	42 (52.5)	31 (50.8)	
Female	127 (52.0)	54 (45.8)	38 (47.5)	30 (49.2)	
BMI					0.355
Range	18.30–63.30	14.50–46.60	17.60–42.40	21.50–51.90	
Mean ± SD	26.87 ± 4.96	25.97 ± 4.098	26.86 ± 4.99	27.39 ± 5.00	
Fitzpatrick skin type					0.167
I	15 (6.5)	2 (1.8)	3 (4.0)	4 (7.0)	
II	90 (38.8)	49 (44.5)	33 (44.0)	28 (49.1)	
III	113 (48.7)	58 (52.7)	37 (49.3)	22 (38.6)	
IV	14 (6.0)	1 (0.9)	2 (2.7)	3 (5.3)	
Fitzpatrick score					0.927
Range	1–26	3–25	4–25	2–25	
Mean ± SD	14.32 ± 4.62	14.67 ± 4.12	14.32 ± 4.56	14.03 ± 5.13	
Immunosuppression					**0.018**
No	186 (76.2)	96 (81.4)	71 (88.8)	55 (90.2)	
Yes	58 (23.8)	22 (18.6)	9 (11.3)	6 (9.8)	
PAASI					**< 0.001**
Range	0–280	0–181	0–222	0–189	
Mean ± SD	75.05 ± 41.94	66.22 ± 43.87	54.48 ± 45.96	51.76 ± 43.58	
Lifetime sunburns					0.106
Rarely	87 (35.8)	35 (29.9)	19 (24.1)	12 (20.3)	
Occasionally	108 (44.4)	50 (42.7)	43 (54.4)	34 (57.6)	
Often	48 (19.8)	32 (27.4)	17(21.5)	13 (22.0)	
Childhood sunburns					0.844
Rarely	93 (38.6)	42 (36.5)	29 (37.2)	19 (32.2)	
Occasionally	95 (39.4)	45 (39.1)	34 (43.6)	29 (49.2)	
Often	53 (22.0)	28 (24.3)	15 (19.2)	11 (18.6)	
Sunbathing					0.137
Very rarely	51 (21.2)	12 (10.3)	17 (21.8)	11 (19.0)	
Occasionally	87 (36.1)	50 (42.7)	23 (29.5)	25 (43.1)	
Often/very often	103 (42.7)	55 (47.0)	38 (48.7)	22 (37.9)	
Occupation type					**0.014**
Indoor	148 (61.4)	90 (76.3)	58 (73.4)	43 (74.1)	
Mixed/outdoor	93 (38.6)	28 (23.7)	21 (26.6)	15 (25.9)	
Haemoglobin					0.052
Range	110–205	113–169	119–182	105–170	
Mean ± SD	140.43 ± 12.43	141.73 ± 11.09	144.38 ± 11.00	140.53 ± 13.46	
Leukocyte count					0.075
Range	2.6–14.1	2.4–10.7	3.1–10.7	3.2–11.2	
Mean ± SD	6.49 ± 1.80	6.16 ± 1.61	5.93 ± 1.52	6.16 ± 1.85	
Platelet count					0.421
Range	77–490	69–451	114–397	146–425	
Mean ± SD	242.67 ± 58.67	245.96 ± 62.40	246.01 ± 55.69	257.35 ± 62.03	
Lymphocyte count					0.246
Range	0.6–6.1	0.8–5.7	0.7–3.4	0.7–3.4	
Mean ± SD	1.92 ± 0.71	1.83 ± 0.75	1.79 ± 0.52	1.79 ± 0.69	
Neutrophil count					0.367
Range	1.6–10.6	1.1–8.2	1.3–8.0	1.1–9.5	
Mean ± SD	3.95 ± 1.54	3.73 ± 1.24	3.59 ± 1.37	3.82 ± 1.69	
Eosinophil count					0.144
Range	0.0–2.7	0.0–0.8	0.0–0.9	0.0–1.6	
Mean ± SD	0.19 ± 0.21	0.16 ± 0.11	0.17 ± 0.14	0.17 ± 0.22	
Platelet‐to‐lymphocyte ratio					**0.037**
Range	40.53–421.43	32.86–278.33	70.71–565.71	62.65–358.89	
Mean ± SD	140.91 ± 59.26	148.41 ± 51.53	147.97 ± 61.96	166.05 ± 74.49	
Neutrophil‐to‐lymphocyte ratio					0.757
Range	0.64–14.00	0.53–6.27	0.66–10.57	0.61–9.25	
Mean ± SD	2.36 ± 1.58	2.32 ± 1.12	2.20 ± 1.33	2.52 ± 1.82	
Eosinophil‐to‐lymphocyte ratio					0.406
Range	0.00–1.00	0.00–0.62	0.00–0.43	0.00–1.78	
Mean ± SD	0.11 ± 0.09	0.09 ± 0.08	0.10 ± 0.080	0.12 ± 0.24	
SII					0.618
Range	140.80–4130.0	52.57–1637.87	163.43–4186.29	136.89–2763.44	
Mean ± SD	568.74 ± 413.56	568.31 ± 305.05	563.40 ± 498.86	661.27 ± 537.44	
Past or present extracutaneous malignancy					0.264
No	203 (83.2)	103 (87.3)	71 (88.8)	56 (91.8)	
Yes	41 (16.8)	15 (12.7)	9 (11.3)	5 (8.2)	
Number of AKs					**< 0.001**
0	100 (41.0)	71 (60.2)	60 (75.0)	51 (85.0)	
1–3	75 (30.7)	25 (21.2)	11 (13.8)	6 (10.0)	
4–10	39 (16.0)	13 (11.0)	8 (10.0)	2 (3.3)	
Over 10	30 (12.3)	9 (7.6)	1 (1.3)	1 (1.7)	
Family history of CMM					**0.012**
No	219 (89.8)	104 (88.1)	63 (78.8)	47 (77.0)	
Yes	25 (10.2)	14 (11.9)	17 (21.3)	14 (23.0)	

*Note:* Continuous variables were tested with the Kruskal‐Wallis test, and categorical variables were tested using the *χ* ^2^ or Fisher's exact test. Statistically significant values are shown in bold.

Abbreviations: AK, actinic keratosis; BMI, body mass index; CMM, cutaneous malignant melanoma; PAASI, PhotoAging area and severity index; SD, standard deviation; SII, systemic immune‐inflammation index.

Regarding blood cells and biomarkers (Table 1), no significant differences were found between the nevus groups for NLR, ELR, SII, Hb, thrombocytes, and leukocytes. However, a statistically significant difference in PLR values was observed, with higher PLR values corresponding to a greater number of MN (140.9, 148.4, 148.0, 166.1, respectively; *p* = 0.04).

Due to a notable difference in immunosuppression status across nevus groups, the analysis was also performed for the 408 immunocompetent subjects only (Table 2). The results remained consistent, with significant differences in mean age, PAASI and AKs, all showing a decreasing trend with increasing MN count (*p* < 0.001), while PLR increased (*p* = 0.01). NLR, ELR, SII, and blood cell counts showed no significant variation between groups.

**Table 2:** Comparison of immunocompetent subjects (*n* = 408) by nevus count categories.

	Observed nevus count				*p* value
	0–20 nevi	21–50 nevi	51–100 nevi	Over 100 nevi	
	*n* = 186	*n* = 96	*n* = 71	*n* = 55	
Age					**< 0.001**
Range	32–79	24–79	24–79	21–77	
Mean ± SD	69.27 ± 8.16	61.01 ± 13.22	56.38 ± 15.95	53.41 ± 14.46	
PAASI					**< 0.001**
Range	0–280	0–181	0–222	0–189	
Mean ± SD	78.00 ± 41.73	63.78 ± 42.09	57.56 ± 46.59	50.77 ± 43.05	
Platelet‐to‐lymphocyte ratio					**0.012**
Range	43.61–320.77	53.68–278.33	70.71–225.33	62.65–325.00	
Mean ± SD	130.87 ± 47.91	143.59 ± 48.14	142.54 ± 40.91	155.05 ± 62.29	
Number of AKs					**< 0.001**
0	63 (33.9)	55 (57.3)	53 (74.6)	46 (85.2)	
1–3	67 (36.0)	22 (22.9)	9 (12.7)	5 (9.3)	
4–10	29 (15.6)	11 (11.5)	8 (11.3)	2 (3.7)	
Over 10	27 (14.5)	8 (8.3)	1 (1.4)	1 (1.9)	

*Note:* Continuous variables were tested using the Kruskal‐Wallis test, and categorical variables were tested using the *χ* ^2^ or Fisher's exact test. Statistically significant values are presented in bold.

Abbreviations: AK, actinic keratosis; PAASI, PhotoAging area and severity index; SD, standard deviation.

### 3.2 Correlation Analysis Between Platelet‐to‐Lymphocyte Ratio and Other Variables

In 505 subjects (Supporting Information S1: Table 1), a statistically significant but weak inverse correlation was found between PLR and age (*r* = −0.107, *p* = 0.02). A similar weak inverse correlation was observed between PLR and body mass index (BMI) (*r* = −0.190, *p* < 0.001), which was confined to immunocompetent subjects (*r* = −0.194, *p* < 0.001). Regarding the Fitzpatrick score, a very weak positive correlation was found (*r* = 0.090, *p* = 0.05). No correlation was observed between PLR and PAASI. In summary, although some statistically significant correlations were observed, their strength was weak and of limited clinical relevance. Additionally, PLR was significantly higher in immunosuppressed individuals (180.67 ± 87.21) than immunocompetent subjects (138.80 ± 49.36) (*p* < 0.001).

### 3.3 Comparison Between Subjects With Atypical Mole Syndrome and Controls

Of the 505 subjects, 69 were diagnosed with AMS, with 35 having 51–100 MN and 34 having more than 100 MN. The results are shown in Table 3. Compared to control subjects (*n* = 436), a higher proportion of AMS subjects were male (*p* = 0.009), and they were significantly younger (*p* < 0.001), had higher BMI (*p* = 0.02), and more frequently reported a family history of CMM (*p* < 0.001). They also showed lower PAASI scores (*p* < 0.001), lower number of AKs (*p* < 0.001), and higher hemoglobin levels (*p* < 0.001). Additionally, PLR was significantly elevated in AMS subjects (162.46 ± 65.57) compared to controls (144.56 ± 59.78, *p* = 0.028). No significant differences were seen in other CBC markers.

**Table 3:** Comparison of subjects with atypical mole syndrome (*n* = 69) and subjects without atypical mole syndrome (*n* = 436).

	Subjects without atypical mole syndrome	Subjects with atypical mole syndrome	*p* value
	*n* = 436	*n* = 69	
Age, years			**< 0.001**
Range	22–79	21–79	
Mean ± SD	64.14 ± 12.42	52.46 ± 14.85	
Sex			**0.009**
Male	211 (48.4)	45 (65.2)	
Female	225 (51.6)	24 (34.8)	
BMI			**0.022**
Range	14.50–63.30	17.60–51.90	
Mean ± SD	26.55 ± 4.71	27.83 ± 5.11	
Immunosuppression			0.091
No	348 (79.8)	61 (88.4)	
Yes	88 (20.2)	8 (11.6)	
PAASI score			**< 0.001**
Range	0–280	0–189	
Mean ± SD	69.84 ± 44.06	49.33 ± 39.69	
Fitzpatrick skin type			0.066
I	20 (4.9)	4 (6.1)	
II	163 (39.9)	37 (56.1)	
III	208 (50.9)	23 (34.8)	
IV	18 (4.4)	2 (3.0)	
Fitzpatrick score			0.339
Range	1–26	2–24	
Mean ± SD	14.47 ± 4.50	13.82 ± 4.93	
Number of AKs			**< 0.001**
0	224 (51.5)	58 (84.1)	
1–3	108 (24.8)	9 (13.0)	
4–10	61 (14.0)	2 (2.9)	
Over 10	42 (9.7)	0 (0.0)	
Family history of CMM			**< 0.001**
No	385 (88.3)	50 (72.5)	
Yes	51 (11.7)	19 (27.5)	
Smoking history			0.060
Never	232 (53.8)	45 (66.2)	
≤ 20 years	142 (32.9)	20 (29.4)	
> 21 years	57 (13.2)	3 (4.4)	
Alcohol use			0.116
never	58 (13.7)	4 (6.1)	
≤ 1×/month	142 (33.6)	31 (47.0)	
3–4×/month	120 (28.4)	17 (25.8)	
1–2×/week or more	103 (24.3)	14 (21.2)	
Past or present extracutaneous malignancy			0.181
No	372 (85.3)	63 (91.3)	
Yes	64 (14.7)	6 (8.7)	
Occupation type			0.930
Mainly outdoor	32 (7.4)	5 (7.5)	
Mainly indoor	293 (68.0)	47 (70.1)	
Mixed	106 (24.6)	15 (22.4)	
Sunbathing			**0.035**
Very rarely	77 (17.9)	14 (20.9)	
Occasionally	153 (35.7)	33 (49.3)	
Often/very often	199 (46.4)	20 (29.9)	
Lifetime sunburns			0.570
Rarely	136 (31.5)	19 (27.9)	
Occasionally	199 (46.1)	36 (52.9)	
Often	97 (22.5)	13 (19.1)	
Childhood sunburns			**0.047**
Rarely	153 (35.8)	30 (44.1)	
Occasionally	174 (40.7)	31 (45.6)	
Often	100 (23.4)	7 (10.3)	
History of UV phototherapy			0.335
No	373 (90.1)	61 (93.8)	
Yes	41 (9.9)	4 (6.2)	
History of tanning bed use			0.413
No	303 (70.1)	51 (75.0)	
Yes	129 (29.9)	17 (25.0)	
Haemoglobin			**< 0.001**
Range	105‐205	114‐182	
Mean ± SD	140.69 ± 11.83	146.03 ± 12.60	
Leukocyte count			0.546
Range	2.4–14.1	3.2–10.7	
Mean ± SD	6.30 ± 1.73	6.18 ± 1.74	
Platelet count			0.174
Range	69–490	147–425	
Mean ± SD	244.00 ± 59.63	254.49 ± 58.41	
Lymphocyte count			0.222
Range	0.5–6.1	0.7–3.2	
Mean ± SD	1.88 ± 0.71	1.74 ± 0.57	
Neutrophil count			0.614
Range	1.1–10.6	1.6–8.0	
Mean ± SD	3.81 ± 1.46	3.91 ± 1.52	
Eosinophil count			0.066
Range	0.0–2.7	0.0–1.6	
Mean ± SD	0.18 ± 0.17	0.17 ± 0.21	
Platelet‐to‐lymphocyte ratio			**0.028**
Range	32.86–565.71	63.67–358.89	
Mean ± SD	144.56 ± 59.78	162.46 ± 65.57	
Neutrophil‐to‐lymphocyte ratio			0.190
Range	0.53–14.00	0.86–8.56	
Mean ± SD	2.33 ± 1.51	2.47 ± 1.34	
Eosinophil‐to‐lymphocyte ratio			0.173
Range	0.00–1.00	0.00–1.78	
Mean ± SD	0.10 ± 0.087	0.11 ± 0.23	
SII			0.122
Range	52.57–4186.29	188.57–2763.44	
Mean ± SD	568.27 ± 418.64	649.74 ± 442.98	

*Note:* Continuous variables were tested using the Mann‐Whitney test, and categorical variables were tested using the *χ* ^2^ or Fisher's exact test. Statistically significant values are shown in bold.

Abbreviations: AK, actinic keratosis; BMI, body mass index; CMM, cutaneous malignant melanoma; PAASI, PhotoAging area and severity index; SD, standard deviation; SII, systemic immune‐inflammation index.

### 3.4 Logistic Regression Analyses for Melanocytic Nevi (Subjects With > 50 Nevi vs. ≤ 50 Nevi)

**Figure 1:**
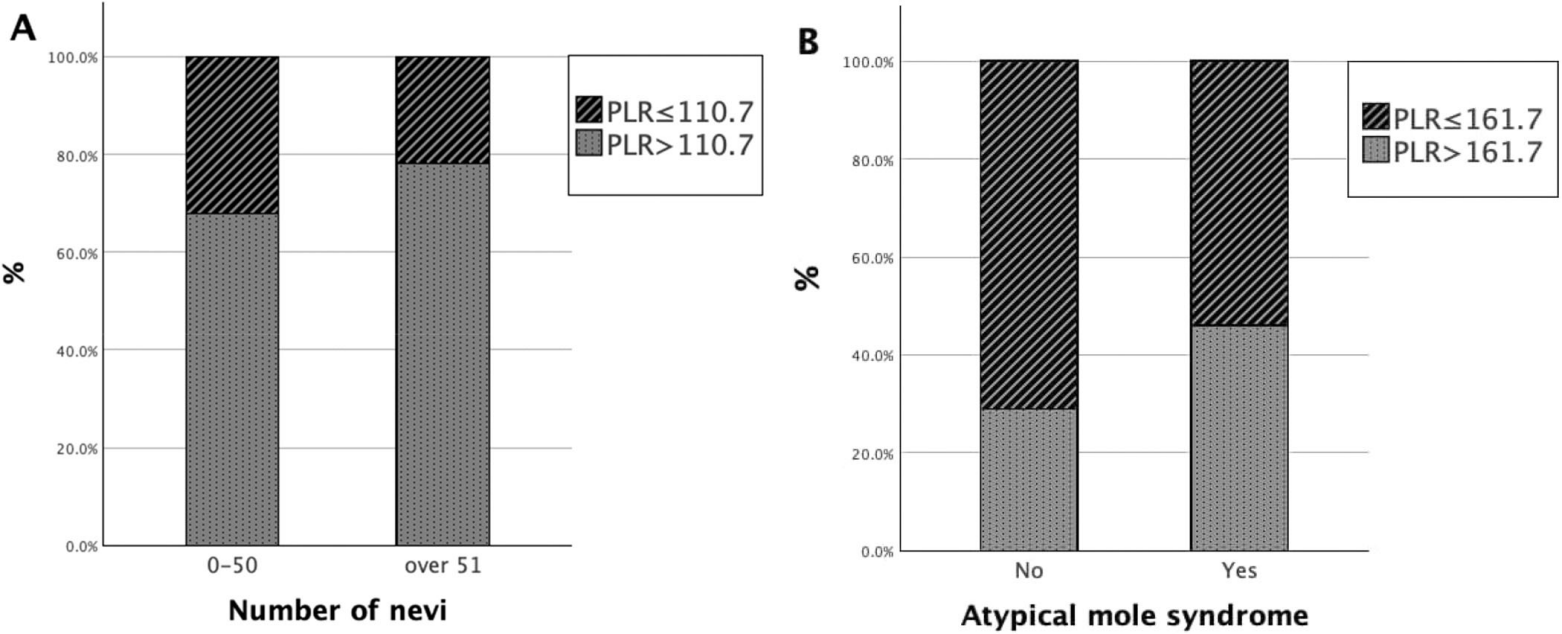
Proportion of subjects with high and low platelet‐to‐lymphocyte ratio (PLR) in different study groups. Proportion of subjects with high and low platelet‐to‐lymphocyte ratio (PLR) according to (A) nevus count and (B) atypical mole syndrome status. Panel A shows the percentage of subjects with PLR above and below the cutoff value (110.7) in groups with 0–50 nevi and ≥ 51 nevi. Panel B shows the percentage of subjects with PLR above and below the cutoff value (161.7) in individuals without and with atypical mole syndrome. Abbreviation: PLR, platelet‐to‐lymphocyte ratio.

Risk factors for having more than 50 MN, compared to 50 or fewer, were assessed using binary logistic regression analyses (Table 4). The optimal PLR cutoff value was set at 110.7 according to the maximum Youden's index derived from the ROC‐curve (Figure 1). In univariable regression analyses, older age, immunosuppression status, PAASI, and AKs were related to lower risk, whereas a positive family history of CMM and lifetime sunburns were related to increased risk. A high PLR value (> 110.7) was also associated with elevated risk (OR 1.696, *p* = 0.03). In multivariable analysis (Table 4), older age, female gender, immunosuppression status, and higher number of AKs remained independently associated with decreased odds of having more than 50 MN, while higher BMI was connected to increased risk, as was having high PLR (OR 2.015, *p* = 0.01). A ROC‐curve was formed, and the AUC‐value for this model was 0.798.

**Table 4:** Binary logistic regression analysis of potential predictors for having > 50 nevi (*n* = 132) compared to ≤ 50 nevi (*n* = 349).

	Simple OR	*p* value	95% CI	Multivariable OR	*p* value	95% CI
Age	0.940	**< 0.001**	0.926–0.955	0.942	**< 0.001**	0.922–0.964
Sex					**0.015**	0.339–0.892
Male	1 (ref)			1 (ref)		
Female	0.932	0.721	0.631–1.375	0.550		
BMI	1.022	0.286	0.982–1.064	1.060	**0.016**	1.011–1.111
Immunosuppression					**< 0.001**	0.118–0.481
No	1 (ref)			1 (ref)		
Yes	0.420	**0.004**	0.233–0.757	0.238		
PAASI	0.989	**< 0.001**	0.984–0.994	1.006	0.084	0.999–1.012
Lifetime sunburns						
Rarely	1 (ref)			1 (ref)		
Occasionally	1.918	**0.008**	1.188–3.097	1.552	0.125	0.885–2.721
Often	1.476	0.185	0.830–2.625	0.968	0.987	0.498–1.954
Platelet‐to‐lymphocyte ratio					**0.013**	1.159–3.501
Low (≤ 110.7)	1 (ref)			1 (ref)		
High (> 110.7)	1.696	**0.027**	1.061–2.709	2.015		
Number of AKs						
0	1 (ref)			1 (ref)		
1–3	0.262	**< 0.001**	0.149–0.462	0.366	**0.003**	0.188–0.710
4–10	0.296	< **0.001**	0.145–0.607	0.409	**0.044**	0.171–0.977
Over 10	0.079	< **0.001**	0.019–0.334	0.141	**0.011**	0.031–0.643
Family history of CMM					0.190	0.811–2.870
No	1 (ref)			1 (ref)		
Yes	2.334	**0.001**	1.389–3.921	1.526		

*Note:* The multivariable odds ratios (ORs) are adjusted for all covariates presented in the table. The statistically significant values are shown in bold. Ref = reference group. Abbreviations: AK, actinic keratosis; BMI, body mass index; CI, confidence interval; CMM, cutaneous malignant melanoma; PAASI, PhotoAging Area and Severity Index.

### 3.5 Logistic Regression Analyses for Atypical Mole Syndrome (AMS vs. Controls)

For comparing subjects with AMS and those without, the optimal cutoff value for PLR was set at 161.7 (Figure 1). In univariable regression analyses assessing risk factors for AMS (Table 5), higher BMI, alcohol use, a family history of CMM, and elevated PLR were associated with increased risk. In contrast, older age, female gender, immunosuppression status, PAASI, presence of AKs, long smoking history, and childhood sunburns were connected to lower risk. In multivariable analysis (Table 5), higher BMI and a family history of CMM remained independently associated with an increased risk for AMS, while older age, female gender, immunosuppression status, and presence of multiple AKs were associated with decreased risk. Elevated PLR (> 161.7) also significantly increased the odds of AMS (OR 4.092, *p* < 0.001). The ROC‐curve yielded an AUC‐value of 0.875 for this model.

**Table 5:** Binary logistic regression analysis of potential predictors for atypical mole syndrome (*n* = 69) compared to subjects without atypical mole syndrome (*n* = 436).

	Simple OR	*p*‐value	95% CI	Multivariable OR	*p*‐value	95% CI
Age, years	0.945	**< 0.001**	0.929–0.962	0.945	**< 0.001**	0.916–0.975
Sex					**< 0.001**	0.061–0.285
Male	1 (ref)			1 (ref)		
Female	0.500	**0.010**	0.294–0.850	0.132		
BMI	1.050	**0.044**	1.001–1.102	1.128	**< 0.001**	1.061–1.199
Immunosuppression					**0.013**	0.103–0.767
No	1 (ref)			1 (ref)		
Yes	0.519	0.096	0.239–1.124	0.281		
PAASI score	0.987	**< 0.001**	0.980–0.994	1.007	0.114	0.998–1.016
Number of AKs						
0	1 (ref)			1 (ref)		
1‐3	0.322	**0.003**	0.154–0.674	0.452	0.099	0.176–1.160
Over 4	0.075	< **0.001**	0.018–0.313	0.036	**0.003**	0.004–0.318
Family history of CMM					**0.011**	1.285–6.693
No	1 (ref)			1 (ref)		
Yes	2.869	< **0.001**	1.569‐5.246	2.933		
Smoking history						
Never	1 (ref)			1 (ref)		
≤ 20 years	0.726	0.268	0.412–1.280	0.586	0.167	0.275–1.250
> 21 years	0.271	**0.034**	0.081–0.905	0.410	0.193	0.107–1.571
Alcohol use						
never	1 (ref)			1 (ref)		
≤ 1×/month	3.165	**0.037**	1.069–9.370	0.985	0.983	0.250–3.881
3–4×/month	2.054	0.213	0.661–6.381	0.498	0.340	0.119–2.084
1–2×/week or more	1.971	0.250	0.620–6.267	0.510	0.363	0.119–2.179
Childhood sunburns						
Rarely	1 (ref)			1 (ref)		
Occasionally	0.909	0.731	0.526–1.570	1.101	0.795	0.533–2.273
Often	0.357	**0.019**	0.151–0.844	0.365	0.066	0.125–1.070
Platelet‐to‐lymphocyte ratio						2.012–8.323
Low (≤ 161.7)	1 (ref)			1 (ref)		
High (> 161.7)	2.087	**0.007**	1.219–3.575	4.092	**< 0.001**	

*Note:* The multivariable odds ratios (ORs) are adjusted for all covariates presented in the table. The statistically significant values are shown in bold. Ref = reference group. Abbreviations: AK, actinic keratosis; BMI, body mass index; CI, confidence interval; CMM, cutaneous malignant melanoma; PAASI, PhotoAging area and severity index.

## 4 Discussion

To our knowledge, no previous studies have explored the association between MN and systemic inflammatory biomarkers. Therefore, the essential new finding of this cross‐sectional study is the observed association between PLR and both MN count and AMS, whereas other markers—including NLR, ELR, and SII—showed no such association. A notable strength of this study lies in the comprehensive assessment of MN and AMN across the entire body, including both UVR‐exposed areas and typically sun‐protected regions. Additionally, the clinical examinations were conducted by experienced dermatologists, reducing the likelihood of misclassifying seborrheic keratoses, solar lentigines, or other non‐melanocytic lesions.

However, this study has several limitations. The use of a selected high‐risk population limits the generalizability of the findings. The relatively small sample size and cross‐sectional design restrict the ability to assess causality or long‐term clinical relevance. Moreover, CBC‐derived ratios are nonspecific markers of inflammation and can be influenced by various confounding factors, such as infections and medications, which were not controlled for in this study. To confirm these findings and reduce potential biases, larger prospective studies with robust methodology are warranted.

The formation and involution of MN involve a complex interplay of genetic, environmental, and host‐related factors [17, 45, 46]. Classically, MN have been thought to develop during the first two or three decades of life, and show a tendency to regress after the sixth decade [47, 48]. However, these observations of decreasing MN counts have primarily been made in cross‐sectional studies. More recent longitudinal studies have yielded conflicting results, showing that the total MN count can also increase in older subjects [49, 50, 51]. Consistent with prior cross‐sectional studies, MN counts in this study declined with age. Longitudinal data from this cohort may reveal whether the MN count truly decreases with age or if other factors, such as selection bias, could account for this observation.

Furthermore, MN exhibit dynamic behavior not only in their number but also in their size and morphology. After an initial mutation triggers the formation of MN, melanocytes go through a brief period of growth and then enter a stable, senescence‐like phase, although a small portion of melanocytes seems to retain the ability to proliferate [52, 53]. This limited proliferation is balanced by simultaneous immune‐mediated removal of some MN cells, resulting in most MN maintaining a relatively stable size [52]. Dermoscopic changes also occur in MN over time; however, the frequency of these changes declines with advancing age [54]. Other well‐established factors influencing the size and number of MN in adults, beyond age, include immunosuppression, UVR exposure, and physiological states like pregnancy [20, 23, 45, 55]. Interestingly, in contrast to prior literature, immunosuppression was less common among subjects with higher MN counts or AMS in this cohort, which may reflect selection bias or differences in clinical characteristics.

The relationship between UVR and MN appears to be two‐sided. UVR affects MN density both through direct effects on melanocytes and via indirect immunosuppressive effects on the cutaneous microenvironment [56]. UVR exposure has been associated with increased MN formation, particularly following intermittent, intense sun exposure, whereas the effects of chronic, cumulative sun exposure appear to be less significant [15, 16, 45, 55, 57]. Furthermore, MN involution is likely influenced by UVR, with more pronounced involution occurring in areas of chronic exposure, although genetic factors also contribute to this process [45, 58]. In addition, AKs and chronic sun damage have been linked to lower MN count [59, 60, 61]. The current findings align with these observations, as the indicators of photodamage, PAASI and AKs, were clearly decreased in individuals with higher MN counts, who also more frequently reported an indoor occupation. However, no differences were observed between groups regarding recreational sun exposure or sunburn history. Interestingly, genetic factors may have a greater influence on MN density in countries with lower levels of UVR, such as Finland, where the present study cohort was based [62].

The pathophysiology of sporadic AMS remains poorly understood. Epidemiological studies have demonstrated an association between the AMS phenotype and sun exposure [58]. However, in the present study, subjects with AMS reported fewer childhood sunburns and less sunbathing compared to those without AMS. Given that AMS can manifest in either a sporadic or hereditary form, and these were not distinguished in this study, it is plausible that the influence of sun exposure was outweighed by a stronger genetic predisposition in this cohort. Moreover, AMS subjects more often reported a family history of CMM, which may have contributed to increased risk awareness and adoption of sun‐protective behaviors. This interpretation is supported by their lower photodamage, as measured by AKs and PAASI. Previous studies have shown that intermittent, intense UVR exposure, rather than cumulative long‐term sun exposure, is more strongly associated with the number of AMN [57, 63]. Furthermore, AMN are often present in sites with minimal to no UVR exposure, such as the buttocks, which implies that AMN can develop without any direct UVR exposure [64]. In the present study, the AMS phenotype was more common among male subjects, which is consistent with previous studies [13]. AMN have also been associated with fair skin types [13]. Although a higher proportion of AMS subjects in the current cohort exhibited Fitzpatrick skin types I or II, the difference was not statistically significant.

An increase in BMI was associated with both elevated MN counts and the presence of AMS. Previous studies have connected height, rather than weight, with higher MN counts and increased CMM risk, possibly due to common mechanisms of growth and reduced senescence, such as telomere length [65, 66, 67].

In the context of cancer prognosis, CBC‐derived ratios are thought to reflect both cancer‐associated inflammation and reduced antitumor immunity [32, 34, 68]. While only a few studies have investigated the relationship between blood cell parameters or CBC‐derived ratios and cancer risk, emerging evidence suggests that alterations in these markers may occur years before a cancer diagnosis [69, 70, 71, 72, 73, 74]. However, most of these studies have not specifically addressed CMM risk. Future research is needed to determine whether similar changes in blood biomarkers precede the diagnosis of CMM. A better understanding of CBC‐derived ratio levels in individuals with multiple MN or AMS—both key risk factors for CMM—could improve risk assessment in high‐risk populations.

A potential link between elevated PLR and the presence of numerous MN and AMS may reflect the immune system's role in regulating melanocytic proliferation or inflammation associated with MN. Several biological observations support the connection between MN and the immune system. For example, eruptive nevi, where multiple MN appear following immunosuppressive therapy or conditions and subsequently regress after the discontinuation of treatment, highlight the crucial role of a functional immune system in controlling melanocytic proliferation [21, 75, 76]. Additionally, histologically dysplastic nevi often exhibit signs of a host immune response, such as chronic inflammatory infiltrates, suggesting an interaction between the neoplastic cells of dysplastic nevi and the immune system [4, 77, 78]. Similar inflammatory changes are sometimes observed in common MN as well [78, 79]. Furthermore, decreased nevus counts have been reported in bone marrow transplant recipients with chronic graft‐versus‐host disease, suggesting a potential impact of the inflammatory environment on melanocytes [80]. Similar observations have also been made in patients with atopic dermatitis [26].

Another example of the complex interaction between MN and the immune system is the phenomenon of halo nevi—benign MN surrounded by a visible depigmented ring, which ultimately leads to the complete regression of the nevus. Histologically, halo nevi initially exhibit a dense inflammatory infiltrate surrounding the central nests of nevus cells, eventually resulting in the total absence of melanocytes in the affected area [81]. This phenomenon is thought to represent an immunoprotective response against skin neoplasia; however, the exact trigger for the immune system's activation against typically benign nevi remains unclear [81]. Halo nevi are most common in children and adolescents, likely due to more efficient immunosurveillance against melanocytes [82]. In contrast, in adults, the presence of halo nevi is often associated with concurrent malignancy or immunotherapy [83]. In patients with CMM treated with immune checkpoint inhibitors, cutaneous depigmentation, known as vitiligo‐like leukoderma, is sometimes observed [84, 85, 86]. This phenomenon has been linked to a more favorable prognosis, likely due to a more potent immune response targeting antigens shared by both melanoma cells and normal melanocytes [84, 85, 86]. Collectively, these observations highlight the intricate relationship between MN and the immune system, suggesting that immune surveillance, along with the surrounding inflammatory environment, plays a crucial role in regulating melanocyte proliferation.

PLR values were significantly higher among immunosuppressed subjects compared to immunocompetent individuals. This elevation may reflect immune system deviations associated with immunosuppression, although immune‐mediated diseases themselves could also have influenced the results. Notably, PLR did not show correlation with PAASI in this cohort. UVR is known to induce direct DNA damage, inflammation, and the release of immunosuppressive mediators in the skin [87, 88]. Interestingly, its immunomodulatory effects extend beyond the skin and can be observed systemically [56, 89]. Given this, a correlation between PLR and PAASI, an index designed to reflect chronic actinic damage, might have been expected; however, none was found. Furthermore, while PLR showed a statistically significant inverse correlation with BMI, the magnitude of this correlation was very weak and not clinically significant, despite the established link between obesity and low‐level systemic inflammation.

Most studies on CBC‐derived biomarkers use cutoff values based on the study population itself, with varying methodologies employed to determine these thresholds. For melanoma prognosis, for example, cutoff values for PLR vary widely, ranging from 99 to 206 [31, 34]. However, studies establishing true reference values for these biomarkers are scarce. It is well‐documented that reference values vary by age and sex; yet the few available studies on this topic report conflicting findings, likely due to differences in study populations [44, 90, 91, 92]. To facilitate the reliable clinical use of these biomarkers, large‐scale studies in healthy populations are required to establish baseline reference values. Furthermore, understanding confounding factors beyond acute infections and medications is essential for integrating these biomarkers into clinical prognostication frameworks. For instance, dermatological conditions are rarely accounted for in study designs and are likewise seldom used as exclusion criteria, despite their potential to confound systemic inflammatory markers. The current study connects PLR with the presence of multiple MN and AMS, which could serve as confounding factors in CBC ratio‐related CMM research, given their high prevalence among melanoma patients.

In summary, this study identifies a novel association between elevated PLR values and both high MN counts and the AMS phenotype. This finding contributes to the growing body of evidence suggesting that CBC‐derived ratios may reflect biological processes relevant to cancer prognosis and, potentially, cancer susceptibility. Understanding the role of these markers in individuals at increased risk of CMM is essential for their accurate application in melanoma prognosis and risk stratification. However, larger longitudinal studies are needed to confirm the clinical significance and causality of these findings, and to further elucidate the interplay between immune‐mediated processes and melanocytic lesion development.

## Author Contributions

**Reetta Nevakivi:** data curation, formal analysis, investigation, and writing – original draft. **Hanna Siiskonen:** conceptualization, data curation, methodology, supervision, writing – review and editing. **Salla Haimakainen:** conceptualization, data curation, methodology, supervision, writing – review and editing. **Ilkka T. Harvima:** conceptualization, data curation, funding acquisition, investigation, methodology, project administration, supervision, writing – review and editing.

## Ethics Statement

The study was approved by the Ethics Committee of Kuopio University Hospital (approval number: 71/2017) and conducted in accordance with the Declaration of Helsinki.

## Consent

All participants provided written informed consent prior to enrollment. All data collected was managed in compliance with the General Data Protection Regulation of the European Union.

## Conflicts of Interest

The authors declare no conflicts of interest.

## Transparency Statement

The lead author Reetta Nevakivi, affirms that this manuscript is an honest, accurate, and transparent account of the study being reported; that no important aspects of the study have been omitted; and that any discrepancies from the study as planned (and, if relevant, registered) have been explained.

## Supporting information

Supmat.docx.

## Data Availability

The data sets analyzed during the current study are not publicly available due to the risk of compromising patient safety and privacy but are available from the corresponding author on reasonable request.
