# Supplementary material for: A Cross‐Sectional Study of Platelet‐to‐Lymphocyte Ratio in Relation to Pigment Cell Nevi and Atypical Mole Syndrome
Source: Health Sci Rep. 2025 Jun 11;8(6):e70888. doi: 10.1002/hsr2.70888 (PMC12153012; doi:10.1002/hsr2.70888)
Supplement: Supplementary file 1 — Supmat.docx. [file HSR2-8-e70888-s001.docx]

Supplementary Table 1. Correlation of Platelet-to-Lymphocyte Ratio with Other Parameters in Subjects with and without Immunosuppression

|  | All subjects  n=505 | Correlation | p-value | Non-IS subjects  n= 409 | Correlation | p-value | IS  subjects  n=96 | Correlation | p-value | IS vs. non-IS  p-value |
| --- | --- | --- | --- | --- | --- | --- | --- | --- | --- | --- |
| PLR  Mean±SD | 146.88±60.78 | | | 138.80±49.36 | | | 180.67±87.21 | | | **<0.001** |
| Age  Mean±SD | 62.54±13.38 | -0.107 | **0.018** | 62.97±13.47 | -0.073 | 0.148 | 60.69±12.92 | -0.153 | 0.140 | **0.043** |
| BMI  Mean±SD | 26.72±4.78 | -0.190 | **<0.001** | 26.93±4.76 | -0.194 | **<0.001** | 25.84±4.79 | -0.034 | 0.747 | **0.020** |
| Fitzpatrick score  Mean±SD | 14.38±4.56 | 0.090 | **0.048** | 13.98±4.50 | 0.042 | 0.411 | 16.06±4.43 | 0.087 | 0.405 | **0.000** |
| PAASI  Mean±SD | 67.03±44.02 | -0.028 | 0.542 | 67.54±43.90 | -0.005 | 0.919 | 64.85±44.68 | -0.103 | 0.322 | 0.471 |

The significance of the correlations was assessed using the Spearman correlation test. Differences between IS and non-IS subjects were tested using the Mann-Whitney test. Statistically significant values are presented in bold. *Abbreviations:* IS, Immunosuppressed; PLR, Platelet-to-lymphocyte ratio; BMI, body mass index; PAASI, PhotoAging Area and Severity Index; SD, standard deviation.
